# A Novel *GCK* Large Genomic Rearrangement in a Patient with MODY-2 Detected by Clinical Exome Sequencing

**DOI:** 10.3390/genes13112104

**Published:** 2022-11-13

**Authors:** Paola Concolino, Linda Tartaglione, Elisa De Paolis, Cinzia Carrozza, Andrea Urbani, Angelo Minucci, Dario Pitocco, Concetta Santonocito

**Affiliations:** 1 Clinical Chemistry, Biochemistry and Molecular Biology Operations (UOC), Fondazione Policlinico Universitario A. Gemelli IRCCS, 00165 Rome, Italy; 2 Unit of Diabetes Care, Fondazione Policlinico Universitario A. Gemelli IRCCS, 00165 Rome, Italy; 3 Department of Basic Biotechnological Sciences, Intensivological and Perioperative Clinics, Catholic University of Sacred Heart, 00165 Rome, Italy; 4 Departmental Unit of Molecular and Genomic Diagnostics, Fondazione Policlinico Universitario A. Gemelli IRCCS, 00165 Rome, Italy

**Keywords:** maturity-onset diabetes of the young, MODY, *GCK*, clinical exome solution, copy number variation, MLPA, MODY2

## Abstract

Maturity-onset diabetes of the young (MODY) is a rare form of non-autoimmune diabetes with an autosomal dominant inheritance. To date, 14 genes have been reported as genetic basis of MODY. *GCK* gene, encoding the glucokinase enzyme, was the first MODY gene to be identified. *GCK* heterozygous inactivating variants cause the GCK-MODY or MODY2 subtype. However, partial or whole gene deletions have been rarely identified, showing it to be a rare cause of *GCK*-MODY. We reported the molecular evaluation of a Ukrainian patient with clinical diagnosis of MODY2. We performed the Next generation sequencing of the clinical exome using the Clinical Exome Solution^®^ kit (SOPHiA Genetics), followed by the design of a 14 genes virtual panel related to the suggestive diagnosis of MODY. Bioinformatics analysis was performed using the SOPHiA DDM platform (SOPHiA Genetics). The SALSA MLPA kit for MODY (MRC-Holland) was used for relative quantification of GCK exons. From the molecular evaluation, no pathogenic sequence variants were detected in the investigated genes. Copy Number Variation analysis was able to identify a large deletion involving the last three exons of the *GCK* gene. This result was confirmed by MLPA. To the best of our knowledge, the identified rearrangement has never been reported in the literature.

## 1. Introduction

Maturity-onset diabetes of the young (MODY) is a rare, monogenic form of non-insulin-dependent diabetes that classically presents in individual with non-ketotic hyperglycaemia and an autosomal dominant inheritance pattern. The age of onset is typically younger than 25 years of age with a strong suggestive family history of diabetes [1]. Prevalence is estimated to be about 1/10,000 in adults and 1/23,000 in children, even if it can be assumed that most cases are missed due to the overlapping of its clinical features with those of type 1 and type 2 diabetes [2].

Over the last two decades, more than 1000 genetic mutations were identified in at least 14 MODY-related genes, defining different disease subtypes (i.e., MODY 1-MODY 14) [3]. Among these, the genes *HNF1A* (MODY3) and *HNF4A* (MODY1), along with *GCK* gene (MODY2), account for approximately 95% of all identified MODY cases [4]. Additionally, the different MODY main subtypes can be further sub-classified in relation to the specific molecular defects affecting the enzyme function, the protein folding, the ion channel functions, or the signal transduction [5].

Given its overall rare occurrence and the wide clinical manifestations, misdiagnosed MODY cases are not uncommon events. At the same time, the prompt identification of such patients represents a critical improvement of their management. For individuals with MODY, genetic testing should be appropriately performed given that it could guide treatment options, comorbidities clinical evaluation, and family members’ risks assessment. In this context, the cost-effectiveness and the overall availability of MODY molecular testing have increased over the years. The advent of high-throughput Next-generation sequencing (NGS) technologies changed the screening strategies enabling simultaneous analysis of a panel of genes at a lower cost and improved turnaround time [6].

Among the genes involved in the MODY occurrence, impairments in the Glucokinase gene (*GCK*) represents the most common cause of MODY in paediatric cases and the second most common cause in adults, with an estimated prevalence of about 22% in all age groups [7]. The *GCK* gene is located on chromosome 7p15.3–p15.1 and it consists of a total of 12 exons that span 45,168 bp and encode for the 465-amino acid glucokinase enzyme. It is expressed in the pancreas, liver, brain, and endocrine cells of the gut, and performs a crucial role in the regulation of insulin secretion [8]. In the pancreatic β cells, GCK catalyses the first glycolysis reaction with the conversion of glucose to glucose 6-phosphate, which is a key regulator of insulin secretion [9]. While activating mutations in *GCK* can lead to hypoglycemia secondary to constitutive hyperinsulinism [10], inactivating mutations result in hyperglycemic conditions. Heterozygous inactivating defects in *GCK* gene cause the autosomal dominant subtype of MODY known as GCK-MODY or MODY2 (MODY2, OMIM# 125851), characterized by mild fasting hyperglycemia. Interestingly, compound heterozygous and homozygous inactivating *GCK* variants are related to permanent neonatal diabetes mellitus [11]. The MODY2 condition is present at birth but it remains often subclinical, with a late identification in the adult life [12]. This MODY subtype is often suspected in cases of incidental asymptomatic mild hyperglycemia without pancreatic autoimmunity [13]. Usually, MODY2 does not require specific treatment options as it leads to a mild fasting hyperglycaemia that is not associated with significant complications [14,15].

To date, more than 900 *GCK* pathogenic variants have been reported (https://www.hgmd.cf.ac.uk/ac/gene.php?gene=GCK, accessed on 10 October 2022), mainly including single-nucleotide variants (SNV) and small insertion/deletions (indels). Partial or whole gene deletions have been identified in a small number of patients and these seem to represent a rare cause of *GCK*-MODY [5].

Traditional Sanger sequencing is not optimized to detect large-scale Copy number variations (CNVs) and this makes it necessary to use a secondary technique in parallel, such as Multiplex Ligation-dependent Probe Amplification (MLPA), to correctly identify the presence of large genomic rearrangements (LGRs). In this context, MLPA is considered the gold standard technique for CNV evaluation. At the same time, the current improvements in bioinformatics evaluation of NGS output data allows an efficient assessment of CNVs. Moreover, high-throughput NGS is successfully applied to the simultaneous analysis of multi-gene panels. In this regard, the strategy adopting “virtual gene panels”, designed using phenotype-based criteria from large clinical exome sequencing (CES) analysis, represent an expanding diagnostic approach in genetic testing [16].

In this study, we reported a novel heterozygous large deletion involving the last three exons of the *GCK* gene, identified by CES followed by virtual panel design in a patient with suspected MODY2. It is, therefore, one of the rare descriptions of MODY2 caused by a CNV event in the *GCK* gene identified using a state-of-the-art molecular approach.

## 2. Materials and Methods

### 2.1. Patient History

The patient is a 31-year-old Caucasian woman of Ukrainian origin diagnosed with diabetes at the age of 21 years old. Her clinical history began at the age of 19 with episodes of recurrent fever accompanied by myalgias. Steroid therapy was started with no benefit. After an episode of glycemic decompensation with polyuria, polydipsia, and unexplained weight loss occurred during the steroid therapy administration, she was set up for insulin therapy with a basal bolus scheme at the age of 20. Re-evaluated at our Unit of Diabetes Care, Fondazione Policlinico Universitario A. Gemelli IRCCS of Rome, in the context of a pre-pregnancy check-up, she reported a familial history of her mother and grandmother with diabetes with the onset at the ages of 31 and 30, respectively. Personal medical records revealed a history of Mediterranean Fever (MEFV), polymyalgia rheumatica, and hyper IgD syndrome. Clinical records and self-monitored blood glucose levels revealed good glycometabolic control, with a fasting plasma glucose level of 6.1–7.1 mmol/L (reference range of 3.6–5.6 mmol/L) and haemoglobin A1c (HbA1c) levels of 43–47 mmol/mol (HbA1c% of 6.0–6.4%) (reference range of 23–41 mmol/mol). Laboratory tests were conducted for pancreatic antibodies, resulting in the absence of glutamic acid decarboxylase (GAD), pancreatic insula (ICA), and anti-Islet Antigen 2 (IA-2) antibodies. At the last evaluation in our Unit (June 2022), she reported the following biochemical parameters: HbA1c of 45 mmol/mol (HbA1c% of 6.3%) and fasting glycaemia of 112 mg/dL. The patient’s current therapy consists of: Colchicine 1 mg/day; Canakinumab 150 mg, 1 fl subcutaneously 1/month; Metformin500, 1 tablet/day; Insulin Glargine 2 UI/day; Insulin aspart 2 UI before breakfast, 2 UI before lunch, and 2 UI before dinner; Rosuvastatin 5 mg 1 tablet/day; and Levotiroxine 50 mcg/day. With the suspicion of a MODY clinical picture, the patient was addressed to molecular evaluation performed at the Departmental Unit of Molecular and Genomic Diagnostics, Fondazione Policlinico Universitario A. Gemelli IRCCS, Rome (Italy).

All procedures performed in this study were in accordance with the ethical standards of the Ethics Committee of Fondazione Policlinico Universitario A. Gemelli IRCCS of Rome. A written informed consent was obtained from the patients for the publication of this report.

### 2.2. Molecular Analysis

A sample of genomic DNA from peripheral blood lymphocytes was obtained with an automatic DNA extractor (QIAcube Connect, QIAGEN) following the indications of the manufacturer. The quantitation of the extracted DNA was performed using the Qubit dsDNA BR fluorimetric assays (Life Technologies). In order to assess the DNA quality, the spectrophotometry NanoDrop^®^ ND-1000 was used. Library preparation, sequencing, and data analysis were performed following validated protocols. The clinical exome was evaluated using the capture-based NGS Clinical Exome Solution^®^ v2 kit (SOPHiA Genetics) that covers the coding regions and splicing junctions of 4490 genes related to rare and inherited conditions. The NGS protocol was then performed in paired-ends reads mode with FastQ only analysis workflow on the Illumina NextSeq550DX^®^ NGS platform (Illumina). The sequencing FastQ data were analysed by Sophia DDM^®^ platform (Sophia Genetics) to detect SNVs, indels, and CNVs. Bioinformatic prediction of CNVs was performed by analyzing the coverage levels of the target regions across samples. The algorithm selects a set of reference samples from the same sequencing run, based on the similarity of coverage patterns. The coverage is normalized by sample and by target region, and CNV prediction is performed using a hidden Markov model algorithm. As a result, the most likely copy number (CN) for each target region is determined. The CNV module detects partial CNVs with the resolution of single exon. The variant calling strategy adopted consists of a filtering of the analysed genes using a phenotype-driver approach on the Sophia DDM^®^ platform. We designed a virtual panel of 14 genes out of those included in the capture kit and associated with MODY, according to Human Phenotype Ontology database (https://hpo.jax.org/app/, accessed on 4 November 2022) and using the entry term HP:0004904: *ABCC8* (NM_000352.6), *APPL1* (NM_012096.3), *BLK* (NM_001330465.2), *CEL* (NM_001807.6), *GCK* (NM_000162.5, LGR_1074t1), *HNF1A* (NM_000545.8), *HNF1B* (NM_000458.4), *HNF4A* (NM_000457.6), *INS* (NM_000207.3), *KCNJ11* (NM_000525.4), *KLF11* (NM_001177716.2), *NEUROD1* (NM_002500.5), *PAX4* (NM_001366110.1), and *PDX1* (NM_000209.4).

The MLPA assay was employed in order to confirm the CNV alterations detected by NGS analysis. In particular, the SALSA MLPA kit for MODY (P241-E1 MODY Mix 1, MRC-Holland) was used for relative quantification of all *GCK* exons (LGR_1074) according to the manufacturer’s instructions. Briefly, 100 ng of genomic DNA was denatured at 98 °C and hybridized with the specific MLPA probe mix at 60 °C overnight. After ligation reaction of annealed probes (54 °C for 15 min), the subsequent PCR reaction was performed for 35 cycles (30 s at 95 °C, 30 s at 60 °C, and 60 s at 72 °C) with a final step of 72 °C for 20 min. In total, 0.7 μL of amplification product were then mixed with 0.4 μL of the GS-600 Size Standard (Applied Biosystems, Whaltam, MA, US), and 10 μL of HiDi Formamide (Applied Biosystems, Whaltam, MA, US), and analyzed using an ABI 3500 Genetic Analyzer (Applied Biosystems, Whaltam, MA, US). The collected data were analyzed using Coffalyser.NET Software (MRC Holland, Amsterdam, Netherlands). Three healthy males and three healthy females were included in the analysis as wild-type controls. CNV expected results that are allele copy numbers of 2 (normal status), 1 (heterozygous deletion), or 3 (heterozygous duplication). The final ratio (FR) of each individual reference probe in the patient samples should be between 0.80 and 1.20 diploid normal copy number. The following cut-off values for the FR of the probes are used: heterozygous deletion, FR between 0.40 and 0.65; and heterozygous duplication, FR between 1.30 and 1.65.

## 3. Results

**Figure 1:**
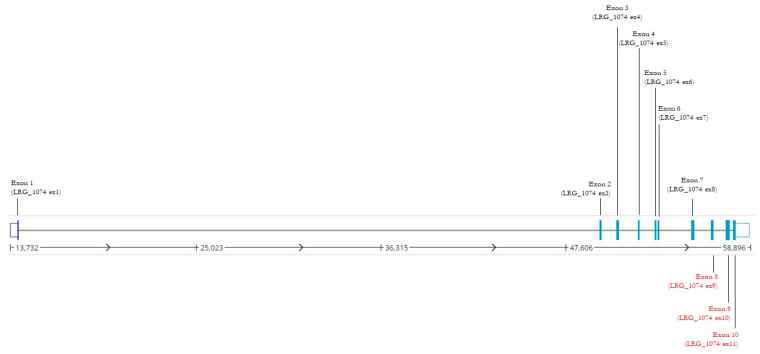
Linear structure of *GCK* gene. For each *GCK* exon, the figure shows the LGR_1074 specific numbering and the NM_000162.5 (LGR_1074t1) transcript specific numbering. Exons involved in the novel rearrangement are reported in red (ftp.ebi.ac.uk/pub/databases/lrgex/LRG_1074.xml, accessed on 4 November 2022).

Sequencing of the entire coding region and exon-intron boundaries of the 14 MODY-related genes failed to detect small indels or point mutations in the affected patient. Likewise, Variants of Unknown Significance suggestive of a phenotype association were not identified in the analysed genes. From the querying of the NGS CNV prediction pipeline, we identified a novel large deletion involving exons 8–10 of the *GCK* gene previously undescribed in the literature or annotated in the main mutational databases (Figure 1).

**Figure 2:**
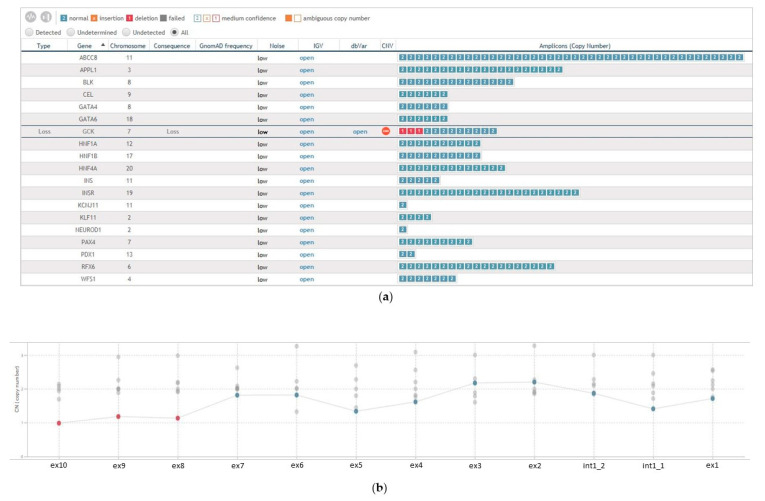
Bioinformatics CNVs prediction of the MODY multi-genes panels performed by Sophia DDM^®^ tool. (**a**) Overview of the copy number analysis of all the 14 MODY-genes with the clear identification of *GCK* exons loss; and (**b**) Copy Number plot of *GCK* gene with indication of exons 8–10 loss. For *GCK*, the exon numbering from the LGR_1074t1 sequence is used.

The bioinformatics pipeline assigned a clear copy number value of 1 to all the three *GCK* exons, suggesting a heterozygous status of the rearrangement (Figure 2, panels A and B).

**Figure 3:**
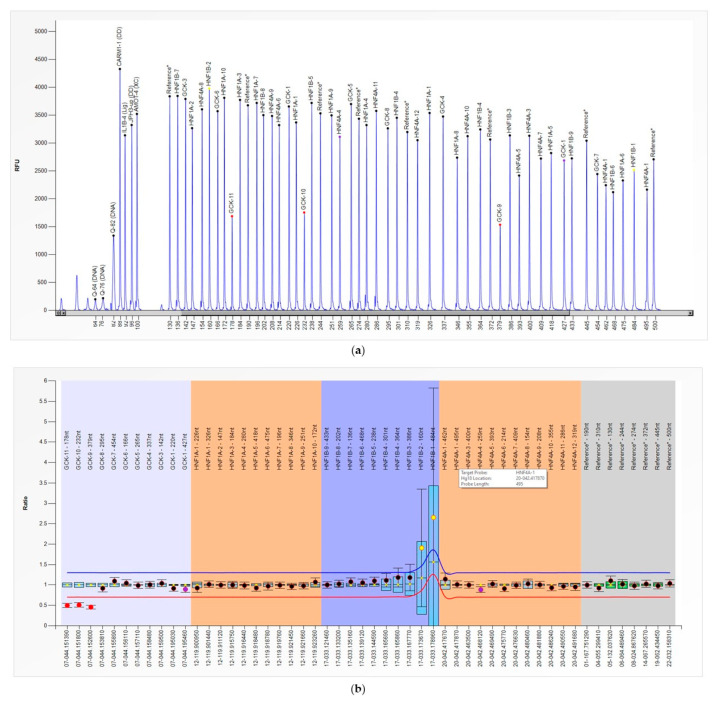
(**a**) MLPA electropherogram from the patient carrying the heterozygous deletion of *GCK* exons 8–10. Specific peaks (*GCK*-9, *GCK*-10, and *GCK*-11) show reduced height. (**b**) MLPA results from comparative analysis experiment obtained by Coffalyser.NET Software (MRC Holland, Amsterdam, Netherlands). The final ratio of exons 8–10 probes was <0.80 (normal range 0.80 < FR < 1.20), indicating a heterozygous deletion. For *GCK*, the exon numbering from the LGR_1074 sequence is used.

The presence of the *GCK* multi-exon deletion was further investigated by the gene dose analysis performed using the MLPA orthogonal method, which confirmed the presence of the heterozygous deletion (Figure 3, panels A and B).

**Figure 4:**
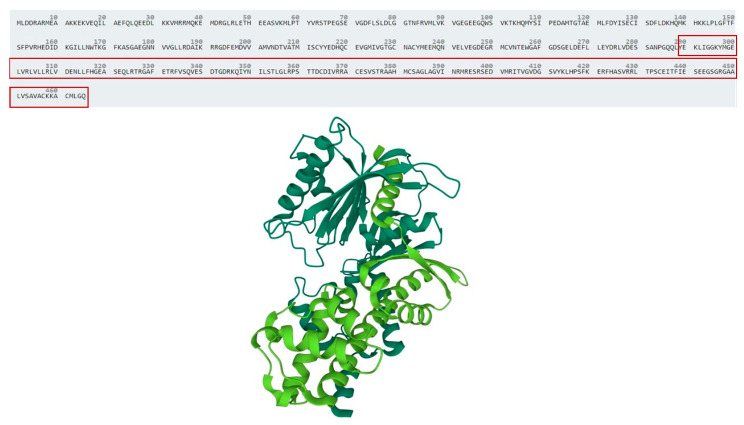
Overall structure of Glucokinase. Figure shows the structure of *GCK* (Hexokinase-4) according to protein databank (PDB) identity 1V4S. Protein region involved in the rearrangement is highlighted in the red box and showed in light green.

The GCK protein (hexokinase IV) is distinctly folded into two domains of unequal size, the small and the large subdomains (aminoacid residues 67–203 and 204–443, respectively) separated by a deep cleft, which forms the active site [17]. Exons 8, 9, and 10 of *GCK* gene are predicted to encode for a relevant part of hexokinase large subdomain, including some key residues of GCK allosteric site (Figure 4) [17,18]. Consequently, the large rearrangement identified in our patients is expected to have a large impact on GCK protein structure and function.

## 4. Discussion

In the present paper, we reported a novel LGRs involving the last three exons of the *GCK* gene identified in a woman affected by MODY2. The patient shows inflammatory and autoimmune diseases, such as Mediterranean Fever (MEFV), polymyalgia rheumatica, and hyper IgD syndrome, but no autoimmunity specific for type 1 diabetes. At the same time, she needs an intensive insulin therapy with a very low requirement—8 Ui of insulin daily—in order to obtain a good metabolic control with good values of Hba1c. She does not have any diabetic complications.

From the molecular evaluation of the patients, a *GCK* deletion was detected by the NGS analysis using the CNV prediction algorithms of SOPHiA DDM software (SOPHiA Genetics), successively confirmed by orthogonal MLPA assay. To date, this represents the only case of large rearrangement identified in our cohort of 31 unrelated patients analyzed for suspected MODY phenotype. In our cohort, causative variants from sequence analysis were identified in 21 probands. Therefore, of the 10 patients in whom no sequence variant was detected, only the described patient (1/10; 10%) was affected by CNV alterations.

*GCK* was the first MODY-related gene to be identified [12,19] and, at present, more than 900 mutations have been described. Missense, nonsense, frameshift, and splice-site variants have been widely reported and are distributed throughout the whole gene sequence, with no mutational hotspots. Differently, partial or whole gene deletions have been identified in a small number of cases and appear to be a rare cause of *GCK*-MODY [20]. In fact, only three types of LGRs, including whole gene deletion, have been reported to date [21,22,23]. Consequently, our findings join this rare group of literature cases.

In particular, as the first *GCK* LGR described, Ellard et al. developed a MLPA assay using synthetic oligonucleotide probes for all the exons of the *GCK*, *HNF1A,* and *HNF4A* genes. The authors analysed 89 unrelated probands in whom no pathogenic sequence variants have been identified by direct sequencing. CNVs consisting in partial or whole gene deletions were identified in 1/29 (3.5%) probands belonging from the *GCK* evaluated sub-group using the *GCK* MLPA assay and in 4/60 (6.7%) of probands of the *HNF1A/4A* cohort using the *HNF1A/4A* MLPA assay [21]. The identified heterozygous *GCK* deletion involved the exon 2 and co-segregated with early-onset diabetes in a UK family, where the young mother and her child were both affected [21]. The described out-of-frame *GCK* deletion was predicted as a result in a premature termination codon and hence to a loss of enzymatic function. The breakpoints for *GCK* exon 2 deletion were adjacent to MIRb and AluSx repetitive elements, suggesting that the rearrangement may have arisen through a non-allelic homologous recombination (NAHR) event [21].

The next year, Garin et al. studied a group of 57 unrelated Spanish patients presenting with a suspected MODY2 phenotype [22]. The 16 index cases without pathogenic variants in the coding region of the *GCK* gene were screened for LGRs by MLPA technique. A heterozygous deletion of the whole *GCK* gene was detected in one case (1/16; 6.2%), and the alteration was also present in the two other affected relatives [22]. Segregation of the gene deletion with the disease in the family was concordant for a causative association. Moreover, the authors reported that the clinical data of the proband was not different from other evaluated cases, indicating that the haploinsufficiency, generating a smaller amount of functional enzyme, does not seem to be more damaging than other types of GCK pathogenic alterations [22].

In 2019, Berberich et al. reported results from re-analysis of 57 individuals clinically suspected to have MODY and were analyzed using a targeted NGS panel (LipidSeq) to assess the presence of pathogenic sequence variants in genes associated with MODY [23]. Since the first-level evaluation of sequence variants failed to detect genetic alterations, NGS data were re-analyzed using the CNV caller tool (VarSeq v1.4.3). Bioinformatics analysis of NGS data detected the same *GCK* heterozygous deletion, encompassing the promoter region and the exon 1 (4763 bp), in two unrelated individuals (2/57; 3.5%). The rearrangement was confirmed in Whole Exome Sequencing-generated data, with identical breakpoints in both patients, subsequently confirmed by Sanger sequencing. Pedigree analysis confirmed co-segregation of the deletion with the phenotype consistent with MODY. In particular, the deletion was detected in the affected mother of the proband 1 and in the affected mother and sister of the proband 2. Interestingly, the clinical presentation in the two *GCK*-MODY probands was similar as both presented fasting hyperglycemia, mild elevations in HbA1c, absence of pancreatic auto-antibodies, and polyuria, which is atypical for *GCK*-MODY. Based on this evidence, the authors speculated that this large-scale deletion may cause a slightly more severe phenotype than other forms of *GCK*-MODY. However, they also reported that both affected mothers and the sister of proband 1 appeared to have only mild phenotypes, concluding that gender differences, associated with phenotype expression and other factors, such as variable penetrance, environmental, or lifestyle, could influence the disease manifestation, explaining the milder phenotype in the carrier females [23]. Other whole gene deletion cases have been recently reported in the literature [24,25,26].

During the last decades, NGS methodologies have become widely used for diagnostic and research purposes. Their application, now common, has increased the chance to identify the molecular and genetic basis of human disorders [27]. Many laboratories had gradually expanded the number of tests offered to patients and, in the meantime, their genes content: starting from molecular test applied to single causative gene, we are now witnessing a move to large disease-targeted multigene panel, even up to WES and whole genome sequencing (WGS) [28]. In this context, an emerging option is the generation of personalized virtual gene panels from the more extensive approach of CES [16]. In fact, one of the major limitations of target NGS multigene panels is the “static” nature of their gene content that prevents the “open” analysis of newly discovered genes, according to the latest scientific knowledge. The molecular approach described here represents an optimized strategy that allows an extensive evaluation of molecular basis of disease. Furthermore, the NGS pipeline described represents a comprehensive analysis that includes the evaluation of large rearrangement from sequencing data. This kind of analysis is rarely applied to MODY-suspected patients in clinical context.

Our study presents some limitations as, at first, the absence of a full segregation analysis of the *GCK* novel molecular alteration due to the unavailability of other family members in Italy. Moreover, after the diagnosis of MODY2, the patient left Italy and we were unable to obtain an additional blood sample to perform the molecular study of the rearrangement breakpoints.

However, we believe that our finding adds relevant information to the rare literature data reporting on GCK LGRs in association with MODY 2 clinical phenotype. Based on the collected data, it is possible to affirm that approximately 5% of patients that test negative to sequencing screening harbour LGRs in GCK gene. However, the overall proportion of MODY cases carrying CNV alterations may become more significant if a CNV investigation was routinely included in the NGS analysis. Here, we showed an extensive NGS approach as the CES, including the full sequencing of more than 4800 genes, is able to clearly identify a three exons deletion event in a disease-causing gene selected via virtual-panel strategy.

## Data Availability

Data are available from the authors upon request.
